# Three-dimensional conformal arc radiotherapy using a C-arm linear accelerator with a computed tomography on-rail system for prostate cancer: clinical outcomes

**DOI:** 10.1186/s13014-015-0515-4

**Published:** 2015-10-12

**Authors:** Atsuhiro Kumabe, Noboru Fukuhara, Takuji Utsunomiya, Takatsugu Kawase, Kunihiro Iwata, Yukinori Okada, Shinya Sutani, Toshio Ohashi, Mototsugu Oya, Naoyuki Shigematsu

**Affiliations:** Department of Radiology, Keio University School of Medicine, 35 Shinanomachi, Shinjuku-ku, Tokyo 160-8582 Japan; Division of Radiation Oncology, Department of Radiology, Sagamihara Kyodo Hospital, 2-8-18 Hashimoto, Midori-ku, Sagamihara, Kanagawa 252-0143 Japan; Department of Urology, Sagamihara Kyodo Hospital, 2-8-18 Hashimoto, Midori-ku, Sagamihara, Kanagawa 252-0143 Japan; Department of Radiation Oncology, National Center for Global Health and Medicine, 1-21-1 Toyama, Shinjuku-ku, Tokyo 162-8655 Japan; Department of Radiology, St. Marianna University School of Medicine, 2-16-1 Sugao, Miyamae-ku, Kawasaki, Kanagawa 216-8511 Japan; Department of Urology, Keio University School of Medicine, 35 Shinanomachi, Shinjuku-ku, Tokyo 160-8582 Japan

**Keywords:** Prostate cancer, Radiotherapy, C-arm

## Abstract

**Background:**

We report the feasibility and treatment outcomes of image-guided three-dimensional conformal arc radiotherapy (3D-CART) using a C-arm linear accelerator with a computed tomography (CT) on-rail system for localized prostate cancer.

**Methods and materials:**

Between 2006 and 2011, 282 consecutive patients with localized prostate cancer were treated with in-room CT-guided 3D-CART. Biochemical failure was defined as a rise of at least 2.0 ng/ml beyond the nadir prostate-specific antigen level. Toxicity was scored according to the National Cancer Institute Common Terminology Criteria for Adverse Events, version 4.0.

**Results:**

A total of 261 patients were analyzed retrospectively (median follow-up: 61.6 months). The median prescribed 3D-CART dose was 82 Gy (2 Gy/fraction, dose range: 78–86 Gy), and 193 of the patients additionally received hormonal therapy. The 5-year overall survival rate was 93.9 %. Among low-, intermediate-, and high-risk patients, 5-year rates of freedom from biochemical failure were 100, 91.5 and 90.3 %, respectively. Rates of grade 2–3 late gastrointestinal and genitourinary toxicities were 2.3 and 11.4 %, respectively. No patient experienced late grade 4 or higher toxicity.

**Conclusions:**

In-room CT-guided 3D-CART was feasible and effective for localized prostate cancer. Treatment outcomes were comparable to those previously reported for intensity-modulated radiotherapy.

## Background

Along with radical prostatectomy and brachytherapy, external beam radiotherapy (EBRT) with or without hormonal therapy is one of the most commonly employed curative treatment options for localized prostate cancer [1–3]. Recent technical developments have allowed conformal treatment to deliver both high-dose radiation to the target volume and reduced radiation to adjacent organs at risk, including the rectum and bladder [4, 5]. By “conformal treatment,” we mean both image-guided radiotherapy (IGRT) and high-technology EBRT, such as three-dimensional conformal radiotherapy (3D-CRT) and intensity-modulated radiotherapy (IMRT). As a consequence of these technical developments, greater effectiveness has been observed in large retrospective studies and several prospective randomized trials of treatment outcomes [6–12]. In fact, dose escalation has improved local control and cause-specific survival [13–15]. Moreover, clinical data from the Fox Chase Cancer Center (Philadelphia, PA, United States) have suggested that a dose–response relationship exists, even in the ultra-high dose range beyond 80 Gy [16]. Further, better disease control was obtained with ultra-high doses, as compared with standard doses [16].

Although relatively steep dose gradients can be applied in IMRT, dose escalation is believed to have limited applicability in 3D-CRT because of treatment-related morbidities. Several studies have shown correlations between higher 3D-CRT doses and increased risks of grade ≥2 late rectal and urinary toxicities [7, 12, 17].

At Sagamihara Kyodo Hospital (Kanagawa, Japan), image-guided three-dimensional conformal arc radiotherapy (3D-CART) has been implemented using a linear accelerator with a computed tomography (CT) on-rail system for localized prostate cancer since 2004. Dose escalation above 78 Gy (median dose: 82 Gy) has been investigated without the use of IMRT since 2006. The CT on-rail system was employed as a tool for IGRT. As compared with IMRT, our treatment demands less time, effort and cost for RT planning and verification because it does not require an inverse-planning approach. To the best of our knowledge, there have not been any published reports regarding the clinical results of 3D-CART for prostate cancer, as applied with similarly high doses and the use of a CT on-rail system as an IGRT tool in Japan. The aim of this retrospective study is to elucidate our method and report the treatment outcomes and toxicities observed in patients who received CT on-rail system-guided 3D-CART as a treatment for prostate cancer.

## Materials and methods

### Patients

Between January 2006 and December 2011, 282 consecutive patients with T1b-T4N0M0 (International Union Against Cancer classification, UICC 2002) histologically proven adenocarcinoma of the prostate were definitively treated with 3D-CART at Sagamihara Kyodo hospital. Patients with castration-resistant disease and patients under treatment for other malignant disease were excluded. Patients whose follow-up period was less than 2 years were also excluded. A total of 261 patients were analyzed retrospectively. This study was approved by the institutional review board of Sagamihara Kyodo hospital.

Patient risk groups were defined following the National Comprehensive Cancer Network (NCCN) Guidelines. Patients were classified as follows: i) low risk: stage ≤ T2a, Gleason score ≤ 6, and prostate-specific antigen (PSA) ≤ 10 ng/ml; ii) high risk: stage ≥ T3, Gleason score ≥8, or PSA >20 ng/ml; iii) intermediate risk: all other patients (including patients with stage T2b-T2c, Gleason score 7, and PSA >10 ng/ml but ≤ 20 ng/ml).

### CT-linear accelerator integrated radiation system at our institution

**Fig. 1:**
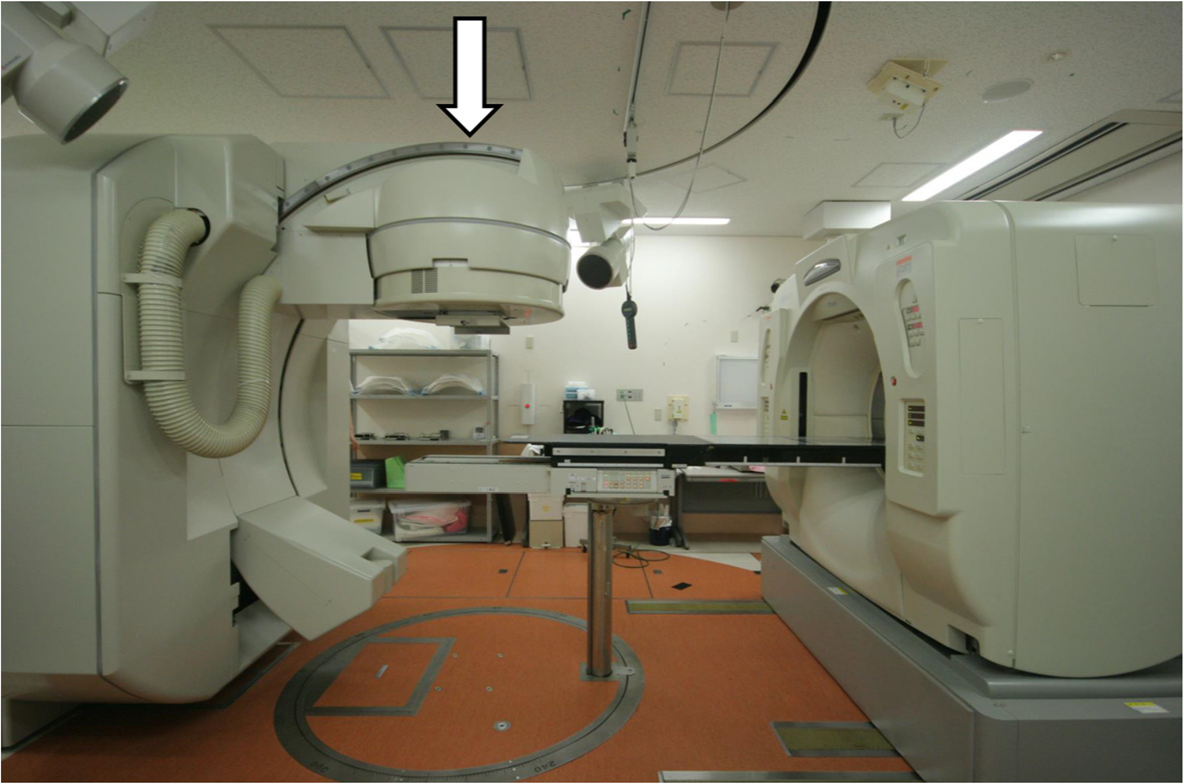
The CT-linac integrated radiation system at Sagamihara Kyodo Hospital. The C-arm mounted linac is indicated by an arrow

Figure 1 shows the CT-linear accelerator (linac) integrated radiation system at Sagamihara Kyodo Hospital. The integrated CT-linac system consists of a linac, a CT scanner, and a treatment couch, which is shared by both the linac and CT scanner. The linac gantry and the CT gantry are set on opposite sides of the treatment couch. By rotating the treatment couch 180°, patients are able to undergo both radiotherapy and CT scanning without having to move by themselves. Radiation treatment simulations and corrections of the patients’ position before daily treatment were performed using this in-room CT system. Radiotherapy was delivered using a C-arm-mounted linac (CRS-6000, Mitsubishi Electric Corporation, Tokyo, Japan). As the name indicates, the C-arm has a C shape, and movement of the gantry head outside the plane of gantry rotation is realized by attaching the head to a C-arm. The head moves on the C-arm rail in a curved path from the vertical position to 60° toward the gantry (at maximum). In general, other similar linacs with a C-arm can deliver radiation and use the C-arm function only for a small field with a maximal length of 3 cm. At our institution, however, the radiation treatment room was broadly shielded with lead, allowing us to use this function for larger treatment fields of up to 40 cm. IMRT cannot be performed with the C-arm linac.

### Treatment

Radiotherapy was delivered daily, five times per week with the 6-MV C-arm linac. Simulation was conducted with the patients in a supine position with a hand-made support under their knees. All patients underwent a treatment planning CT scan with a slice thickness of 2 mm, and the resulting images were transferred to a radiotherapy treatment planning system (Pinnacle,^3^ Philips Medical Systems, Fitchburg, WI, United States). Treatment simulation was performed using the CT-linac integrated system. At the time of CT simulation, patients were asked to void their bowels and to have a comfortably full bladder by urinating and drinking 500 ml of water about one hour before the CT scan. They were asked to do the same before every daily treatment. We did not introduce any fiducial markers into the prostate. Instead, prostate calcifications or the prostate itself was used in setting up patients.

The clinical target volume (CTV) for low-risk patients consisted of only the prostate, the CTV for intermediate-risk patients consisted of the prostate and the proximal half of the seminal vesicles, and the CTV for high-risk patients consisted of the proximal two-thirds of the seminal vesicles. Although there is potential benefit of pelvic nodal irradiation in high-risk patients, the lymph node areas were not included in the CTV, because we were unsure regarding the clinical benefit and the toxicity associated with a high dose of RT of above 78 Gy. The planning target volume (PTV) was obtained by adding a 5-mm margin in all directions, including the posterior aspect of the prostate.

**Fig. 2:**
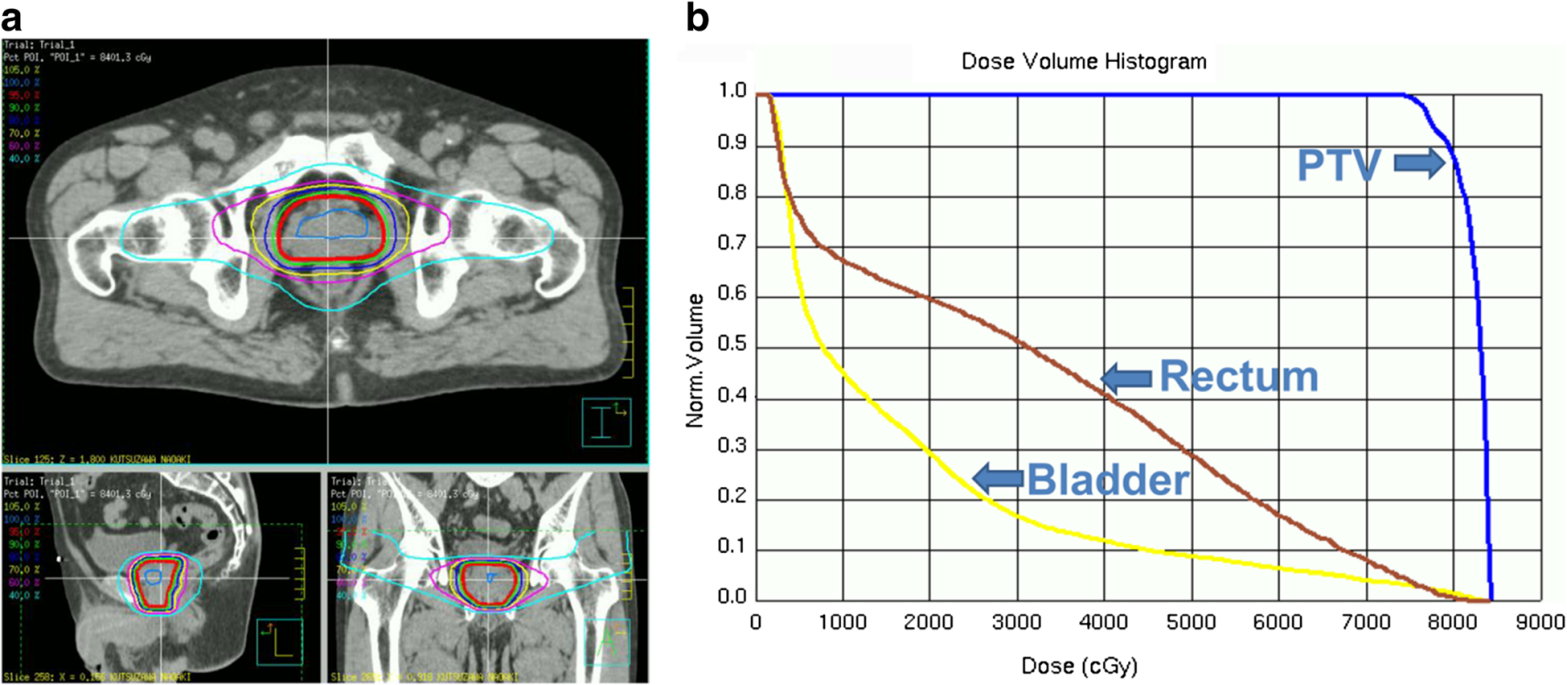
A typical dose distribution (**a**) and dose-volume histogram (**b**) for three-dimensional conformal arc radiotherapy. In the left panel, the thick red line circumscribes 95 % of the prescribed dose. PTV, planning target volume

An additional 5-mm margin was added around the PTV to account for the penumbra. The beam arrangement was as follows: 360°-conformal arc (181° to 180°), right lateral (90°), and left lateral (270°). The lateral two ports were non-coplanar; they were inclined 15° from the same plane, such that the C-arm function of the gantry head could be used to exclude the femoral heads from the treatment field without shifting the treatment couch. These two fields were blocked with a multi-leaf collimator such that the treatment field did not include more than 5 mm of the anterior rectal wall. Figure 2a shows a typical dose distribution, and Fig. 2b shows a dose-volume histogram of 3D-CART for a patient treated to a total dose of 84 Gy. The treatment dose was prescribed to the isocenter. The rectum (from the anal verge to the sigmoid flexure) and the entire bladder were delineated as the organs at risk. Table 1 shows the dose-volume relationships for the PTV, rectum, and bladder.

**Table 1:** Dose-volume relationships for the planning target volume, rectum, and bladder for 261 patients

Patients	IC dose	D95 (PTV)	V80R	V70R	V50R	V80B	V70B	V50B
	(Gy)	(Gy)	(%)	(%)	(%)	(%)	(%)	(%)
median	82	75.9	0.4	6.7	24.5	5.5	12.2	22.2
range	78–86	70.8–80.9	0–3.2	3.0–11.2	12.1–42.2	0–18.9	2.8–30.7	7.8–50.5

*PTV* planning target volume, *IC* isocenter, *D95(PTV)* the dose given to 95 % of the PTV; V80R, V70R, and V50R, percent of the volume of the rectum receiving 80 Gy, 70 Gy, and 50 Gy, respectively; V80B, V70B, and V50B, percent of the volume of the bladder receiving 80 Gy, 70 Gy, and 50 Gy, respectively

Before treatment, each patient underwent a CT examination of the pelvis, and the couch shared by the CT and linac was rotated 180° toward the linac. Subsequently, the patient’s position was corrected according to the image in the setting of the simulation. The CT images taken before treatment and from the simulation were compared side-by-side on the monitor. After imaging performed using in-room CT, image registration was done manually. Patients underwent 39 to 43 additional CT scans throughout the treatment course. With regard to additional doses due to the daily IGRT protocol, the physicians at our department estimated the approximate additional dose received by the patient as 34 cGy over 40 fractions. According to the report from the American Association of Physicists in Medicine Task Group 179 [18], the imaging dose per scan from kV-CBCT ranged from 0.1 to 2 cGy, meaning that the total additional dose for 40 fractions of treatment is expected to range from 4 to 80 cGy. This additional dose using our IGRT method is comparable to the dose from kV-CBCT. Although this additional dose is not negligible, the potential benefit of using the IGRT technique was thought to justify any risks to the patient.

In practice, neoadjuvant androgen deprivation therapy (ADT) was administered to intermediate- and high-risk patients for 6 months. Additionally, adjuvant ADT was administered to high-risk patients for 2–3 years. Because of the retrospective nature of this study, however, the use and period of ADT were determined according to the discretions of the physicians involved and consensus with the patient.

### Follow-up and oncological outcomes

During the course of irradiation, patients were examined weekly by radiation oncologists. Post-treatment clinical follow-up and PSA examinations were generally performed at 3-month intervals for the first 2 years after treatment and every 6 months thereafter. We evaluated freedom from biochemical failure (FFBF) according to the Phoenix criteria; biochemical failure was defined as a rise of at least 2.0 ng/ml beyond the PSA nadir [19]. Toxicity was evaluated according to the National Cancer Institute Common Terminology Criteria for Adverse Events (NCI-CTCAE), version 4.0. Acute toxicities were defined as those occurring during the course of radiotherapy (RT) or within 90 days of its completion. Late toxicities were defined as those occurring thereafter.

### Statistical analysis

According to the clinical outcomes, FFBF and overall survival (OS) were calculated using the Kaplan-Meier method. The statistical significance of differences between survival curves was examined using the log-rank test. All times to events were measured from the initiation of RT. The statistical analysis was performed using SPSS version 22.0 (IBM Corporation, Armonk, NY, United States).

## Results

### Patients

The patients’ characteristics are listed in Table 2, including tumor stage, pretreatment PSA value, Gleason score, risk group, prescribed dose, and the use of hormonal therapy. Of the 261 analyzed patients, 42, 95, and 124 were low-, intermediate-, and high-risk, respectively. The median total dose was 82 Gy (range, 78–86 Gy), and the daily fractional dose was 2 Gy.

**Table 2:** Patient characteristics (*n* = 261)

Characteristics		
Age (years)		
Median	73	
Mean	72.2	
Range	50–85	
Follow-up		
Median (months)	61.6	
	*n*	%
Tumor stage		
≤ T1c, T2a	163	62.5
T2b–T2c	63	24.1
≥ T3a	35	13.4
Gleason score		
≤ 6	79	30.3
7	101	38.7
8–10	81	31.0
PSA		
≤ 10	123	47.1
10–20	67	25.7
20<	71	27.2
NCCN risk group		
Low risk	42	16.1
Intermediate risk	95	36.4
High risk	124	47.5
Prescribed dose (Gy)		
78	22	8.4
80	52	19.9
82	80	30.7
84	85	32.6
86	22	8.4
Hormonal therapy		
(+)	193	73.9
(−)	68	26.1

*NCCN* national comprehensive cancer network, *PSA* prostate specific antigen

### Outcomes

**Fig. 3:**
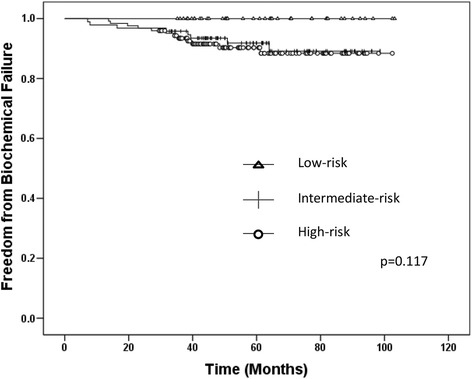
Kaplan-Meier estimates of freedom from biochemical failure, as stratified by NCCN risk. Biochemical failure was defined as a rise of at least 2.0 ng/ml beyond the prostate-specific antigen nadir

**Fig. 4:**
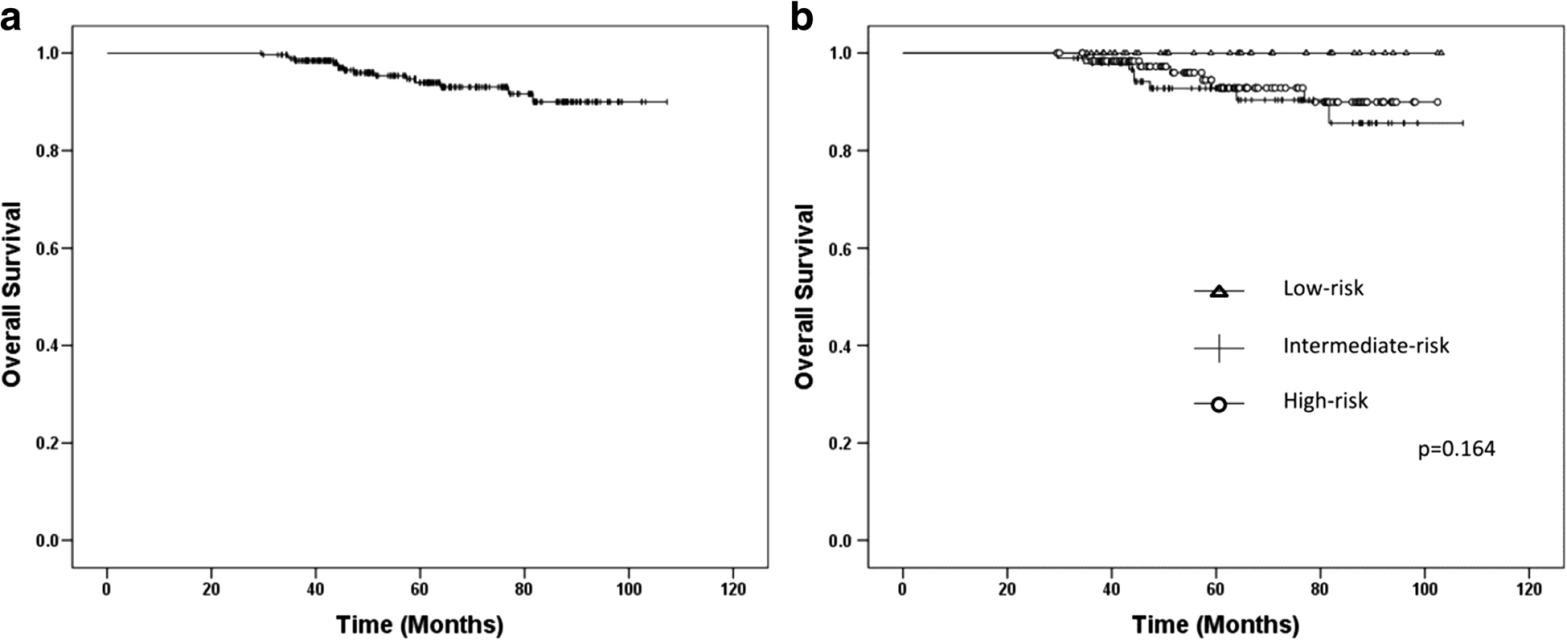
Kaplan-Meier estimates of overall survival for all patients (**a**) and as stratified by NCCN risk (**b**)

The median follow-up period was 61.6 months (range, 29.5–107.3 months). FFBF rates are shown in Fig. 3, as stratified by NCCN risk classification (*p* = 0.117). The 5-year FFBF rates for low-, intermediate- and high-risk patients were 100, 91.8, and 90.3 %, respectively. No patient died of prostate cancer. Fifteen patients died of other causes during the follow-up period. Of these patients, 10 patients succumbed to other malignant diseases, and 5 died as a result of other non-neoplastic diseases (e.g., acute coronary syndrome, pneumonia, and mesenteric thrombosis). Figure 4a shows the OS for all patients (5-year OS rate, 93.9 %) and Fig. 4b shows the OS stratified by NCCN risk classification (*p* = 0.164).

### Toxicity

Acute and late gastrointestinal (GI) and genitourinary (GU) toxicities are summarized in Table 3. Acute GI toxicity was observed in 26.8 % of patients and included anal pain at defecation, anal discomfort, soft stool, and anal bleeding. Acute GU toxicity was more common, occurring in 75.9 % of patients. By 90 days after treatment, 19.2 % patients had developed grade 2 GU urinary symptoms, which were often treated with oral medications (e.g., an alpha blocker or Japanese Kampo). All of the observed acute toxicities were transient. No grade ≥3 acute toxicity was observed.

**Table 3:** Incidences of gastrointestinal and genitourinary acute and late toxicities (*n* = 261)

Grade	GI toxicity *n* (%)		GU toxicity *n* (%)	
	Acute	Late	Acute	Late
1	64 (24.5)	26 (10.0)	148 (56.7)	37 (14.1)
2	6 (2.3)	5 (1.9)	50 (19.2)	27 (10.3)
3	0	1 (0.4)	0	3 (1.1)

*GI* gastrointestinal, *GU* genitourinary

In regard to late GI adverse events, grade 2 and grade 3 toxicities were observed in 1.9 and 0.4 % of patients, respectively. The observed late GI toxicities were rectal bleeding and fecal urgency. One patient developed grade 3 rectal bleeding, which required endoscopic coagulation therapy. In regard to late GU adverse events, grade 2 and grade 3 toxicities were observed in 10.3 and 1.1 % of patients, respectively. The observed late GU toxicities were chronic urinary frequency, urgency, incontinence, urethral stricture, and transient urinary bleeding. Three patients developed grade 3 urethral stricture, which required urethral dilation. No grade ≥4 late toxicity was observed.

## Discussion

This study reports the clinical results of definitive treatment with CT-guided 3D-CART in patients with localized prostate cancer. The clinical outcomes and the treatment-related toxicity profile that we observed during the follow-up period (median, 61.6 months) appeared favorable in comparison with previous reports of IMRT [20–22]. To the best of our knowledge, this is the only report regarding the use of CT on-rail system-guided 3D-CART to deliver high doses (median dose: 82 Gy) for patients with prostate cancer in Japan. Further, in previously published Japanese studies of EBRT for prostate cancer, the prescribed doses have all been under 80 Gy [23–25].

With the development of EBRT, high-dose radiotherapy has become a well-established treatment for localized prostate cancer. In several randomized trials and a meta-analysis, comparisons of high- and low-dose RT have shown that high doses offer significant improvements in biochemical control [11, 12, 26]. Moreover, the findings of some studies have suggested that doses in the ultra-high range (beyond 80 Gy) offer clinical benefit in the form of disease control [16, 20, 27].

Despite the clinical benefits that are offered by dose escalation, the delivery of high-dose RT to the prostate is associated with elevated rates of GI and GU toxicities. The toxicities reported in previous studies of EBRT are summarized in Table 4. Studies that employed 3D-CRT with doses exceeding 74 Gy showed rates of treatment-related grade ≥2 GI and GU toxicities of 5.5–26 and 2.9–28 %, respectively [6–8, 11, 12]. In a randomized trial of 3D-CRT from the M.D. Anderson Cancer Center (Houston, TX, United States), Kuban et al. showed that the rate of grade ≥2 late GI complications was twice as high in patients treated to 78 Gy than in patients treated to 70 Gy (26 % versus 13 %, respectively) [12]. Michalski et al. conducted a multi-institutional dose-escalation phase I–II study of 3D-CRT (RTOG 9406) and reported that grade ≥2 late GI and GU toxicities occurred in 25–26 and 23–28 % of patients, respectively, with a dose of 78 Gy in 39 fractions [8]. Reports of dose escalations above 74 Gy using IMRT have shown rates of grade ≥2 GI and GU toxicities of 2.4–18 and 3.5–22 %, respectively [20–22]. In a retrospective analysis from the Memorial Sloan Kettering Cancer Center (New York, NY, United States), Spratt et al. reported that grade ≥2 late GI and GU toxicities occurred at incidences of 4.4 and 21.1 %, respectively, in patients who had undergone IMRT to a total dose of 86.4 Gy (1.8 Gy/fraction) [20]. Eada et al. showed that grade ≥2 GI and GU toxicities occurred in 2.4 and 3.5 % of patients, respectively, following IMRT with a total dose of 74–78 Gy (2 Gy/fraction) [21]. Comparing the complication rates associated with IMRT and 3D-CRT, GI toxicity rates are generally lower for IMRT [10, 28, 29].

**Table 4:** Overview of reported late toxicities in EBRT for localized prostate cancer

Study (reference)	Patients	Prescription dose	Technique	Posterior margin (other)	GI toxicity (%)		GU toxicity (%)		Grading scale	Follow-up
	(*n*)	(Gy)		(cm)	Grade 2	Grade 3	Grade 2	Grade 3		(months)
Present study	261	78–86	3D-CRT	0.5 (0.5)	1.9	0.4	10.3	1.1	CTCAE4.0	61
RTOG9406 (8)	167	79.2	3D-CRT	0.5–1.0	11–14, Grade 2/3		18–21, Grade 2/3		RTOG	102–110
	220	78			25–26		23–28			71–73
Ikeda et al. [24]	150	74	3D-CRT	0.6 (0.9)	5.5, Grade 2/3		2.9, Grade 2/3		RTOG/EORTC	89
Kuban et al. [12]	151	78	3D-CRT	- (box four field)	19	7	7	3	RTOG/LENT	61
Spratt et al. [20]	1002	86.4	IMRT	0.6 (1.0)	4.4, Grade 2/3		21.1, Grade 2/3		CTCAE4.0	63
Eade et al. [21]	216	74–78	IMRT	0.5–0.6 (0.8)	2.4	0	3	0.5	RTOG	48
De Meerleer et al. [22]	133	74–76	IMRT	0.7 (1.0)	17	1	19	3	RTOG	36

*EBRT* external beam radiotherapy, *3D-CRT* three-dimensional conformal radiotherapy, *IMRT* intensity-modulated radiotherapy, *RTOG* radiation therapy oncology group, *LENT* late effects normal tissue task force, *CTCAE* common terminology criteria for adverse events

In the current study, rates of grade 2 late GI and GU toxicities were 1.9 and 10.3 %, respectively, and rates of grade 3 late GI and GU toxicities were 0.4 and 1.1 %, respectively. There is a striking variation in the toxicity rates that have been reported across different studies. The large variation probably results from differences in treatment methods, radiation doses, and toxicity assessments. Notably, there is no single, standardized tool for measuring treatment-related toxicity. Even when we consider these between-study differences, our results for 3D-CART are comparable to those previously reported for IMRT, although we prescribed high doses (78–86 Gy) and employed a non-IMRT technique. There are several potential explanations for this result, as described below.

First, our approach reduced the volume of the rectum that was exposed to high-dose radiation, as compared with conventional 3D-CRT approaches such as the static five-to-seven port technique. In our 3D-CART approach, the rectal volume that was exposed to high doses (V80R, V70R) compares favorably with that of IMRT (Table 1 and Fig. 2). Our treatment planning method could have contributed to the low frequency of late rectal toxicities.

Second, we used a linac with CT on-rail system as an IGRT tool in daily treatment sessions. Consequently, we were able to confirm that the target was enclosed in the treatment field before every RT session.

Third, we were able to reduce the margins using this IGRT technique. IGRT is believed to be the best method of reducing margins. Sveistrup et al. [30] reported the toxicity rates in patients treated with image-guided IMRT and 3D-CRT without daily image guidance. Three hundred eighty-eight patients were treated at a dose of 78 Gy using IMRT, based on daily image guidance with fiducial markers. Further, 115 patients were treated at a dose of 76 Gy using 3D-CRT without daily image guidance. The 2-year rates of grade ≥2 GI toxicity were 5.8 and 57.3 % in the patients treated with image-guided IMRT and 3D-CRT, respectively (p < 0.001). Regarding GU toxicity, the corresponding values were 29.7 and 41.8 %, respectively (p = 0.011). In a study of IGRT and non-image guided RT, Zelefsky et al. [31] found that IGRT was associated with improved biochemical tumor control in high-risk patients, as well as a lower rate of late urinary toxicity in the total patient cohort.

In the present study, we set a 5-mm margin for the CTV in all directions, which is smaller than the margins reported previously [8, 20, 21, 23, 27]. As a tool for IGRT, the CT on-rail system provides better image quality than is obtainable using cone-beam CT, which is often equipped with the latest linacs. Therefore, we attempted to set up patients using prostate calcifications or the prostate itself as markers in daily IGRT, without implanting artificial fiducial markers (i.e., gold markers).

Fourth, we reduced the prescribed dose by 2–4 Gy in patients who were thought to have possible risk factors for late toxicities (i.e., use of anticoagulants, diabetes, or the occurrence of severe acute toxicities). This therapeutic strategy may also have reduced late toxicities. Considering the risk of toxicities due to the RT, however, 78 Gy remains a high dose for patients with such co-morbidity factors. In hindsight, lower doses should have been used in such patients.

A small PTV margin could leave the target out of the radiation fields, worsening the results of treatment. In our study, however, the 5-year FFBF rates for low-, intermediate-, and high-risk patients were 100, 91.8, and 90.3 %, respectively. Although 15 patients died during the follow-up period, no patient died of prostate cancer and the 5-year OS rate was 93.5 %. These outcomes compare favorably with those of previous reports on IMRT. Our results indicate that high-accuracy positioning with IGRT can allow small PTV margins to be used in EBRT for prostate cancer.

As compared with IMRT, our therapy demands less time and effort for RT planning and verification because it does not require an inverse-planning approach. In order to ensure accurate dose delivery, IMRT requires meticulous implementation and execution with rigorous quality assurance and control. In Japan, two or more full-time radiation oncologists and one or more full-time radiation physicists are needed for IMRT at each radiation therapy facility. However, many institutions do not fulfill these criteria owing to a lack of human resources, and they are therefore not allowed to implement IMRT. At our institution, we performed the 3D-CART technique routinely in daily RT sessions for patients with localized prostate cancer patients, without encountering any difficulty. This method saved time during preparation before RT—in fact, RT began two days after planning CT imaging. Considering the clinical outcomes and ease of preparation before RT, the reported approach may provide another treatment choice for prostate cancer patients.

The key limitations to this study are its retrospective nature and the differences in the toxicity assessment methods that were employed by each radiation oncologist. However, it should also be considered that few patients suffered from severe late treatment-related toxicity during the follow-up period (median duration, 61 months), despite the application of high-dose RT (78–86 Gy). Further, the initial treatment results are comparable to previous reports of IMRT dose escalation studies, although a direct comparison between the 3D-CART approach and IMRT would of course be necessary to reach definitive conclusions. Longer durations of follow-up will be needed to provide a full evaluation of the treatment results, especially for patients who received adjuvant hormonal therapy for long periods. Additionally, given the inclusion of low-risk patients and the absence of deaths from prostate cancer, the study’s results could have been affected by overdiagnosis to some extent, since the outcomes of overdiagnosed patients would not reflect the actual benefits of RT.

## Conclusions

In conclusion, this report confirmed the feasibility and effectiveness of high-dose (≥78 Gy) conformal arc radiotherapy in a large cohort of patients with prostate cancer. The treatment outcomes were comparable to those that have previously been reported for IMRT. In this retrospective study, we found that the 3D-CART approach with IGRT using a CT on-rail system was associated with a low frequency of late rectal toxicities.

## Abbreviations

3D-CART: Three-dimensional conformal arc radiotherapy
3D-CRT: Three-dimensional conformal radiotherapy
ADT: Androgen deprivation therapy
CT: Computed tomography
CTV: Clinical target volume
EBRT: External beam radiotherapy
FFBF: Freedom from biochemical failure
GI: Gastrointestinal
GU: Genitourinary
IGRT: Image-guided radiotherapy
IMRT: Intensity-modulated radiotherapy
Linac: Linear accelerator
OS: Overall survival
PSA: Prostate-specific antigen
PTV: Planning target volume
RT: Radiotherapy
